# Dose-dependent, non-pigmenting fixed drug eruption with eczematous lesions induced by bosutinib: case report

**DOI:** 10.11604/pamj.2023.46.95.42208

**Published:** 2023-12-01

**Authors:** Joohyung Youh, Yasuyuki Yamaguchi, Tsugumichi Kawamura, Daichi Hoshina

**Affiliations:** 1 Department of Dermatology, Hakodate Central General Hospital, Hakodate, Japan,; 2 Sapporo Dermatology Clinic, Sapporo, Japan,; 3 Department of Internal Medicine, Hakodate Central General Hospital, Hakodate, Japan,; 4 Department of Dermatology, Otaru General Hospital, Otaru, Japan

**Keywords:** Bosutinib, fixed drug eruption, non-pigmenting fixed drug eruption, dose-dependent

## Abstract

Bosutinib, widely used as a primary treatment for chronic myeloid leukemia (CML), is known to frequently cause cutaneous drug eruptions. Fixed Drug Eruption (FDE) is common, typically presenting as recurrent lesions that heal with residual hyperpigmentation. Diagnosing FDE, especially Non-Pigmenting Fixed Drug Eruption (NPFDE), is often challenging. A correlation exists between the dosage of certain medications, such as levetiracetam, and the emergence of drug eruptions. This report details a unique case of dose-dependent NPFDE caused by bosutinib. In managing cutaneous drug eruptions, particularly when the causative drug is crucial for treatment, a strategy of tapering the dosage should be considered.

## Introduction

Bosutinib is a first-line treatment for chronic myeloid leukemia (CML) [1]. However, it is frequently associated with cutaneous drug eruptions in patients [2]. Fixed drug eruption (FDE) is a common type of cutaneous drug reaction, characterized by recurrent lesions that heal with residual hyperpigmentation [3]. Diagnosing FDE, especially non-pigmenting FDE (NPFDE), can be challenging. There is a documented correlation between the dosage of the causative drug, such as levetiracetam, and the occurrence of drug eruptions [4]. In this report, we present a rare case of dose-dependent NPFDE attributed to bosutinib.

## Patient and observation

**Patient information:** an 85-year-old Japanese male, undergoing treatment for CML with 200 mg/day of bosutinib, was referred to the dermatology department. He had been diagnosed with CML approximately 10 years prior. His medical history included hypertension, a previous myocardial infarction, insomnia, and constipation. There were no changes in his medication regimen in the four months preceding the referral.

**Figure 1:**
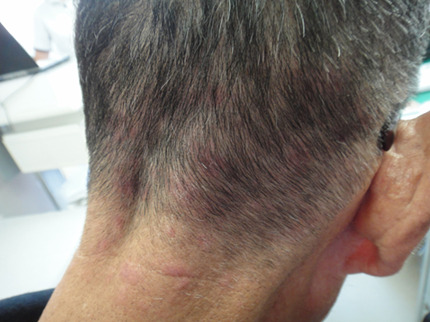
erythematous patches on the lower occipital region and the posterior neck at the first visit

**Clinical findings:** the patient had pruritic, erythematous lesions on his occipital and posterior cervical regions. Examination showed erythematous patches of 1 to 5 cm in diameter on these areas (Figure 1). The lesions were not hyperpigmented and lacked distinct borders.

**Figure 2:**
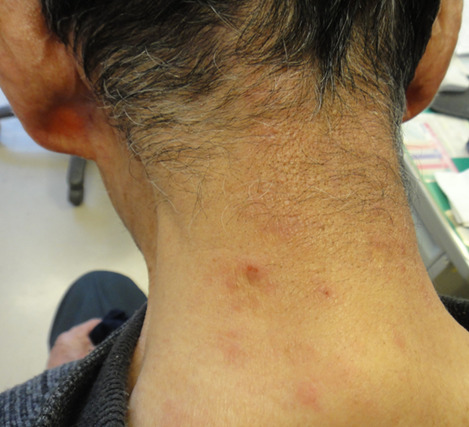
after the temporary cessation of bosutinib due to myalgia, the cutaneous lesions largely resolved

**Figure 3:**
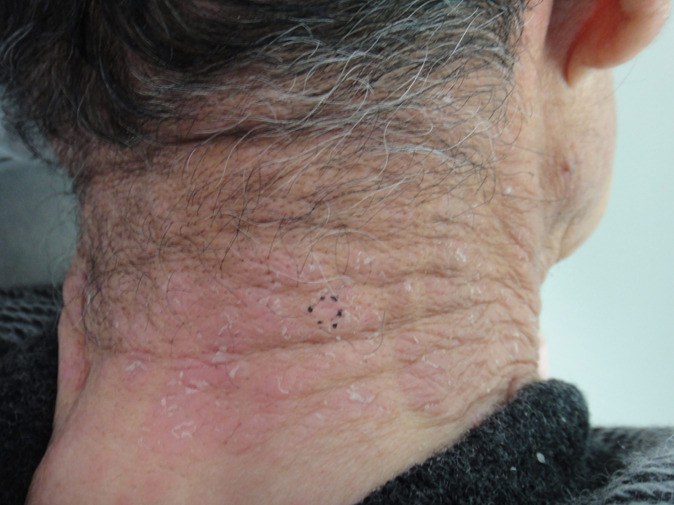
six weeks after the re-administration of bosutinib, cutaneous lesions reappeared and aggravated at the same site

**Timeline of current episode:** bosutinib treatment commenced four months before the dermatology consultation. Skin eruptions appeared three months after initiating the treatment. The lesions significantly subsided following a temporary discontinuation of bosutinib due to myalgia (Figure 2). Six weeks after the myalgia resolved, bosutinib was reintroduced. Subsequently, six weeks later, the lesions reemerged and exacerbated at the same site (Figure 3).

**Figure 4:**
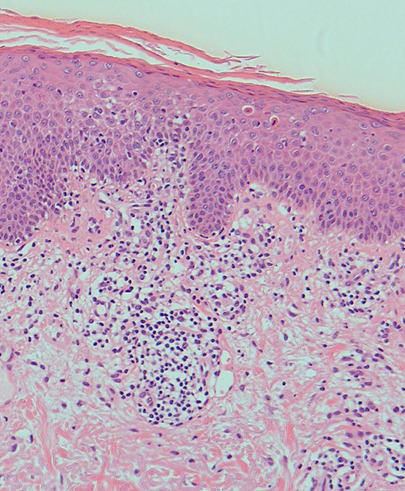
the histopathologic findings: lymphocytes have infiltrated from the dermo-epidermal junction to the superficial dermis; sparse dyskeratotic cells are seen (hematoxylin-eosin stain, x100)

**Diagnostic assessment:** a skin biopsy from an erythematous patch on the posterior neck showed perivascular lymphocytic infiltration extending from the dermo-epidermal junction to the superficial dermis, accompanied by sparse dyskeratotic cells near the junction. Notably, there were no signs of pigmentary incontinence or dermal melanophages (Figure 4).

**Diagnosis:** the clinical presentation suggested a cutaneous drug eruption potentially associated with bosutinib. Although the lesions exhibited an eczematous appearance, histopathological findings, including interface dermatitis and dyskeratotic keratinocytes, were inconsistent with eczema. The morphology and distribution of the lesions aligned with a previous rash but lacked the typical features of FDE. Considering both the clinical progression and histopathological evidence, a diagnosis of NPFDE was suspected.

**Therapeutic intervention:** considering the suspected diagnosis of NPFDE related to bosutinib, the dosage was adjusted to 100 mg daily. Concurrently, treatment was initiated with oral antihistamine (bepotastine besilate) and topical corticosteroid (diflorasone diacetate).

**Follow-up and outcome of interventions:** nine weeks following the dosage reduction, there was a significant subsidence of the cutaneous lesions. The patient has been maintained on this reduced bosutinib dose for six years, with no recurrence of NPFDE.

**Patient perspective:** the patient expressed immense satisfaction and gratitude for the improvement of the cutaneous lesions and pruritus. He was also pleased with the continuation of bosutinib treatment.

**Informed consent:** written informed consent was obtained from the patient for the publication of his data and accompanying images.

## Discussion

Erythema, maculopapular eruptions, acneiform eruptions, and other skin reactions are observed in 20 to 44% of patients treated with bosutinib [2]. The dosage of the causative drug is sometimes associated with the emergence of cutaneous drug eruptions [4].

In our case, re-administering the causative drug led to the recurrence of identical skin lesions at the same site. The absence of hyperpigmented residual lesions after improvement indicated NPFDE. Notably, the skin lesions' response to bosutinib dosage adjustments confirmed a dose-dependent NPFDE. Histopathological findings were consistent with FDE, except for the absence of pigmentary incontinence.

A PubMed search from 1987 to 2022 identified 47 cases of NPFDE. Pseudoephedrine emerged as the most frequent causative agent (10/47 cases). Other associated drugs included amoxicillin (3/47), paracetamol (2/47), dextromethorphan (2/47), ephedrine (2/47), apronal (2/47), ibuprofen (2/47), piroxicam (2/47), mefenamic acid (2/47), and cotrimoxazole (2/47). The variable clinical presentation of NPFDE and its non-hyperpigmented nature suggest potential under-diagnosis. While discontinuing the causative drug is the primary treatment for FDE, dose tapering should be considered, especially when the drug is essential for treatment. This approach is supported by instances of dose-dependent drug eruptions, making dose tapering an effective first-line strategy for managing cutaneous drug eruptions in such scenarios.

## Conclusion

We have presented a rare instance of dose-dependent NPFDE due to bosutinib. To our knowledge, this case represents the first reported instance of NPFDE induced by bosutinib and a dose-dependent FDE. In situations where the causative drug is critical for medical treatment, tapering the dosage should be prioritized as the main strategy.
